# Treating 4,000 diabetic patients in Cambodia, a high-prevalence but resource-limited setting: a 5-year study

**DOI:** 10.1186/1741-7015-7-33

**Published:** 2009-07-14

**Authors:** Marie-Eve Raguenaud, Petros Isaakidis, Tony Reid, Say Chy, Lim Keuky, Gemma Arellano, Wim Van Damme

**Affiliations:** 1 Médecins Sans Frontières, Phnom Penh, Cambodia; 2 Medical Department, Médecins Sans Frontières, Bruxelles, Belgium; 3 Médecins Sans Frontières, Siem Reap, Cambodia; 4 Diabetic Association of Cambodia, Phnom Penh, Cambodia; 5 Institute of Tropical Medicine, Antwerp, Belgium

## Abstract

**Background:**

Despite the worldwide increasing burden of diabetes, there has been no corresponding scale-up of treatment in developing countries and limited evidence of program effectiveness. In 2002, in collaboration with the Ministry of Health of Cambodia, Médecins Sans Frontières initiated an outpatient program of subsidized diabetic care in two hospital-based chronic disease clinics in rural settings. We aimed to describe the outcomes of newly and previously diagnosed diabetic patients enrolled from 2002 to 2008.

**Methods:**

We calculated the mean and proportion of patients who met the recommended treatment targets, and the drop from baseline values for random blood glucose (RBG), hemoglobin A1c (HbA1c), blood pressure (BP), and body mass index (BMI) at regular intervals. Analysis was restricted to patients not lost to follow-up. We used the t test to compare baseline and subsequent paired values.

**Results:**

Of 4404 patients enrolled, 2,872 (65%) were still in care at the time of the study, 24 (0.5%) had died, and 1,508 (34%) were lost tofollow-up. Median age was 53 years, 2,905 (66%) were female and 4,350 (99%) had type 2 diabetes. Median (interquartile range (IQR)) follow-up was 20 months (5 to 39.5 months). A total of 24% (51/210) of patients had a HbA1c concentration of <7% and 35% (709/1,995) had a RBG <145 mg/dl within 1 year. There was a significant drop of 109 mg/dl (95% confidence interval (CI) 103.1 to 114.3) in mean RBG (*P *< 0.001) and a drop of 2.7% (95% CI 2.3 to 3.0) in mean HbA1c (*P *< 0.001) between baseline and month 6. In all, 45% (327/723) and 62% (373/605) of patients with systolic or diastolic hypertension at baseline, respectively, reached = 130/80 mm Hg within 1 year. There was a drop of 13.5 mm Hg (95% CI 12.1 to 14.9) in mean systolic blood pressure (SBP) (*P *< 0.001), and a drop of 11.7 mm Hg (95% CI 10.8 to 12.6) in mean diastolic blood pressure (DBP) (*P *< 0.001) between baseline and month 6. Only 22% (90/401) patients with obesity at baseline lowered their BMI <27.5 kg/m^2 ^after 1 year. Factors associated with loss to follow-up were male sex, age >60 years, living outside the province, normal BMI on admission, high RBG on last visit, and coming late for the last consultation.

**Conclusion:**

Significant and clinically important improvements in glycemia and BP were observed, but a relatively low proportion of diabetic patients reached treatment targets. These results and the high loss to follow-up rate highlight the challenges of delivering diabetic care in rural, resource-limited settings.

## Background

Despite the increasing burden of chronic diseases in the world and the fact that they have now become diseases of poor people in most settings, the needs of these patients have remained largely unmet [1]. Recently, the burden of chronic conditions was assessed in 23 low and middle income countries, showing that they accounted for 50% of the total disease burden in 2005 and were associated with higher estimated death rates in low and middle income countries than in high income countries [2].

The total number of patients with diabetes, one of the most common chronic diseases, is expected to climb from 110 million in 2000 to 317 million by 2030 according to the World Health Organization (WHO) estimates [3]. In Cambodia, recent surveys revealed a diabetes prevalence of 11% in a semi-urban community and an unexpectedly high prevalence of 5% in a relatively poor, traditional, rural community [4]. An estimated 255 000 people live with diabetes today in Cambodia.

Untreated diabetes is associated with uncontrolled hyperglycemia that gives rise to the risk of microvascular damage (retinopathy, nephropathy and neuropathy) and macrovascular complications (ischemic heart disease, stroke and peripheral vascular disease), diminished quality of life and reduced life expectancy. There is now ample evidence that good glycemic control reduces the risk of vascular complications [5-7].

Despite this, access to diabetic care is still limited in developing countries, including Cambodia, and evaluation of care is even more limited. Outcomes of diabetic care management, which generally include measurement of glycemic control and other risk factors such as blood pressure (BP) of large patient cohorts and treatment adherence, is well described in industrialized countries [8-11] whereas there are very few studies of the quality of diabetic care in resource-limited contexts.

In 2002 Médecins Sans Frontières (MSF), in collaboration with the Ministry of Health of Cambodia, initiated a program to provide care for diabetes and hypertension in two public hospital clinics in rural locations. The similarities between the management of these two chronic conditions and HIV/AIDS led MSF to set up a Chronic Disease Clinic offering integrated care for both HIV/AIDS patients and those suffering from diabetes and/or hypertension. This novel experience that demonstrated the feasibility of integrating care for chronic diseases with HIV/AIDS has been published [12]. However, the outcomes of the diabetic patient management have not been fully reported. The purpose of this study is to describe the outcomes of a 5-year diabetic treatment program in a high-prevalence but low-resourcecountry.

## Methods

### Program description

We initiated the diabetic treatment program in March 2002 in Siem Reap province (population 700,000) and in March 2003 in Takeo province (population 800,000). The programs consisted of outpatient clinics at the public referral hospital level and operated similarly in both locations. We provided integrated, patient-centered care (similar to that offered to HIV-positive patients) for those with diabetes, as has been described previously. [12] For diabetes, we enrolled patients with types 1 or 2 and, while we included only patients >15 years old at Siem Reap, patients of all ages were seen at Takeo. Many patients had been previously diagnosed with diabetes, either by a local health provider or a laboratory. Between a quarter and a third of individuals were taking oral antidiabetic drugs at time of first consultation. The clinic staff included general practitioners and nurses trained in diabetes care, drug educators, adherence counselors, a receptionist and support staff to facilitate patient flow. Those same health personnel also attended patients with other chronic conditions (HIV/AIDS, hypertension). MSF subsidized care: patients were required to pay the initial registration fee (US$1.00), a fee for diabetic and antihypertensive drugs (US$1.10 for a 3-month treatment of glibenclamide and US$4.50 for a 3-month treatment of metformin) until mid-2005, and transportation costs. However, from mid-2005 onward, all drugs were free of charge. Transportation costs for patients were never covered by the program.

### Standardized care and follow-up procedures

We defined criteria for the diagnosis of diabetes type 2 as: fasting blood glucose greater than 126 mg/dl on at least two occasions, or random blood glucose (RBG) greater than 200 mg/dl on one occasion with accompanying symptoms and signs of diabetes. We defined diabetes type 1 as child-onset diabetes. Patient admissions were somewhat restricted in 2007 due to high workload.

Advice on exercise and appropriate diet was given to patients on an individual basis. Until 2005 this was provided at the end of the consultation by the doctor, and after 2005 by a nurse in a separate counseling session. However, due to human resources constraints (until 2007 only one nurse was involved in health education), most patients attended only one to two 40-min sessions.

We employed an oral hypoglycemic agent as monotherapy when medical treatment was required: metformin if body mass index (BMI) ≥23 kg/m^2^, and glibenclamide if BMI <23 kg/m^2 ^and/or if there were contraindications to metformin. A second oral agent was added for patients failing to reach glucose control with monotherapy. We reserved insulin therapy for patients with child-onset diabetes, pregnancy, or those with moderate to severe renal impairment. Other non-diabetic drugs such as antihypertensive drugs were used if required clinically. Antihypertensive drugs were started immediately in patients with BP >130/80 and target organ damage. Otherwise, pharmacotherapy was initiated if BP was not controlled (<130/80) 3 months after advice on lifestyle modifications was given to the patient. Angiotensin-converting enzyme (ACE) inhibitors were prescribed as first line antihypertensive therapy. A β blocker was used in patients with cardiac disease. Drugs from other classes, diuretics and calcium inhibitors were added if combination therapy was needed.

We saw patients after treatment initiation every 1 to 2 weeks, then monthly and eventually every 3 months once glycemia was controlled (RBG <200 mg/dl). At each follow-up visit, we measured blood glucose level, weight and BP, and performed clinical screening for complications. BP was measured seated after 5 min rest and involved at least two readings on the same day. We checked for treatment acceptance, tolerance, and adherence with open-ended questions. If drug intake was irregular, counselors provided adherence advice and support. A patient was defined as lost to follow-up (LTFU) if their last contact with the clinic was more than 3 months before and they were not known to be dead or to have transferred out of the area.

We measured glucose levels in capillary blood using a glucometer (OneTouch Basic 200-200; LifeScan, Milpitas, California, USA). In case of very high blood glucose levels, >600 mg/dl, we took venous blood for glucose measurement at the hospital laboratory. Until 2007, due to lack of resources, clinicians generally performed RBG tests in the morning to estimate overall glycemic control and used the cut-off value of 180 mg/dl (10 mmol/l) as an acceptable level [13]. For most patients, baseline RBG was measured between 1.5 to 2 h after breakfast taken at home (typically a light meal high in carbohydrates, since they often had to walk a considerable distance for their appointment). Systematic measurement of hemoglobin A1c (HbA1c) (D-10; high-performance liquid chromatography (HPLC) method) at baseline and on a quarterly basis for monitoring glucose control at the individual level started in 2007. Hence, clinicians used RBG to monitor response to treatment for the majority of the patients for most of their follow-up period.

### Data collection

We recorded clinical information on standardized clinical files specifically designed for diabetic care. Information on all patients was collected prospectively and entered into an electronic medical record software program developed locally, specifically for chronic diseases. Trained personnel extracted clinical, treatment, and laboratory data from individual patient records daily and entered them into the database. A full time data manager routinely checked data entry for accuracy and completeness.

### Statistical analysis

Descriptive statistics (frequency, median, 25th and 75th quartiles, proportion) were used to describe characteristics at admission and status at the end of study period of all patients, both newly and previously diagnosed with diabetes in the program and who came for at least one consultation between March 2002 until mid-2008 in Takeo and Siem Reap clinics.

The analysis of blood sugar, BP and BMI evolution over treatment time was limited to patients not LTFU and with at least baseline and end of follow-up values. We determined the proportion of the patients who met the recommended targets for RBG, HbA1c, BP and BMI at regular intervals after enrolment in the program. For this target analysis, only patients with elevated baseline BMI (≥23.0) were included, for systolic blood pressure (SBP) only patients with elevated baseline SBP (>130 mm Hg) were included, and for diastolic blood pressure (DBP) only patients with elevated baseline DBP (>80 mm Hg) were included. We used the recommended glycemia (RBG <145 mg/dl) and BP targets (130/80) for type 2 diabetic patients proposed by the Asian Pacific type 2 diabetes policy group [14]. For BMI, we referred to the WHO cut-off points for Asian populations: 23 to 27.5 kg/m^2 ^(defined in the study as overweight) and 27.5 kg/m^2 ^or above (defined in the study as obese) [15].

We examined the evolution of these same parameters over time by calculating the mean and standard deviation at regular intervals after treatment initiation. We used the t test to compare the mean differences between paired values at baseline and at month 6 for glucose, BP, and BMI.

We assessed the following potential risk factors for loss to follow-up: age, sex, type of diabetes, year of admission, geographical origin, BMI, baseline BP and last consultation (late or not late). A logistic regression model was performed to assess the association of these potential risk factors with the outcome of lost to follow-up.

We analyzed patient data using Microsoft Excel (Redmond, WA, USA) and STATA software, version 8.2 (STATA, College Station, TX, USA). The analysis considered data from the beginning of the program in 2002 until June 2008. No consent was obtained from individual patients. The use of the medical record data for research purposes was approved by the Ethical Review Board of MSF.

## Results

### Characteristics of study participants and cohort outcomes

**Figure 1:**
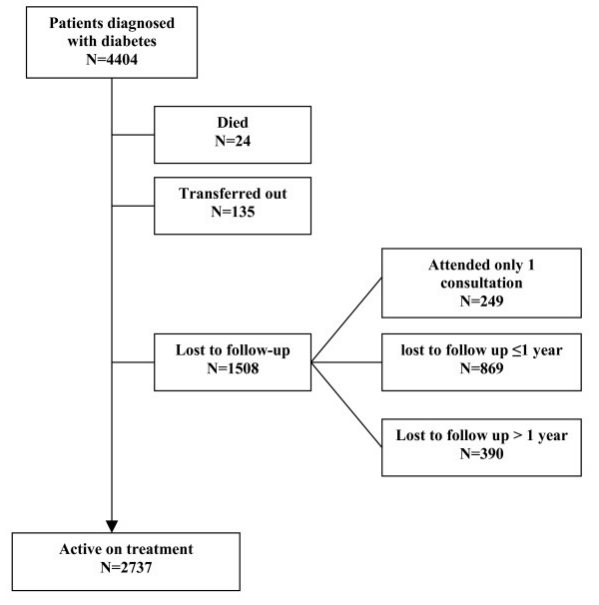
**Outcome of diabetic patients diagnosed in 2002 to 2008 at end of study period (June 2008)**.

A total of 4,404 diabetic patients were registered in the 2 clinics over the 5-year period. Patient characteristics are presented in Table 1. Patients were nearly all diagnosed with type 2 diabetes (99%), were predominantly women, and 64% were ≥50 years old. Over a fifth of patients originated from outside the provinces where the two clinics were located. The median follow-up period per patient was 20 months (interquartile range (IQR) 5 to 39 months). Patients not lost to follow-up underwent an average of 10 consultations per year. At the time of diagnosis, 56% patients were overweight and 16% were obese. Only 4% of patients were prescribed insulin at their last consultation and 41% (1,601/3,952) of patients on oral treatment were prescribed 2 antidiabetic drugs at their last consultation. By the end of the observation period, 2,737 (62%) patients were active on treatment, 24 (0.5%) had died, 135 (3%) were transferred to another clinic, and 1,508 (34%) were LTFU (missed a scheduled appointment by over 3 months) (Figure 1). Most LTFU events (74%) occurred within the first year after diagnosis.

**Table 1:** Characteristics of diabetic patients registered in clinics (March 2002–June 2008)

Characteristic	Value
Total no. of diabetic patients registered	4,404
Type of diabetes, n (%):	
Type 1	54 (1.2%)
Type 2	4,350 (98.8%)
Age in years, median (IQR)	53 (46 to 60)
Age group in years, n (%):	
≥39	438 (10.0%)
40 to 49	1,168 (26.5%)
50 to 59	1,540 (35.0%)
≥60	1,258 (28.6%)
Women, n(%)	2,905 (66.0%)
Origin of patients*, n (%):	
District of clinic	1,724 (41.2%)
Other districts of province	1,550 (37.0%)
Outside province	912 (21.8%)
Random plasma glucose on admission, median (IQR)	272 (196 to 376)
Random plasma glucose <180 mg/dl, n (%)	797/4,095 (19.5%)
HbA1c on admission %, median (IQR) (n = 950)	11.5 (9.1 to 13.5)
HbA1c <7%, n (%)	59/950 (6.2%)
Blood pressure on admission, n (%):	
Systolic ≥140 mm Hg	1,555/3,840 (40.5%)
Diastolic ≥90 mm Hg	1,316/3,840 (34.3%)
BMI on admission, kg/m^2^, median (IQR) (n = 4337):	
All patients	23.5 (20.9 to 26)
Men	23 (20.1 to 26)
Women	23.7 (21.1 to 26.2)
BMI ≥23 kg/m^2^, n (%)	2,433/4,337 (56.1%)
BMI ≥27.5 kg/m^2^, n (%)	686/4,337 (15.8%)
Treatment on last consultation, n (%):	
No antidiabetic drug	91 (2.2%)
Oral antidiabetic drug(s) alone	3,911 (94.1%)
Insulin alone	115 (2.8%)
Oral antidiabetic drug(s) plus insulin	41 (1.0%)
No data	246

*Data on origin available only for patients admitted until February 2008.

BMI = body mass index; HbA1c = glycosylated hemoglobin; IQR = interquartile range.

### Glucose, BP, and weight control

**Figure 2:**
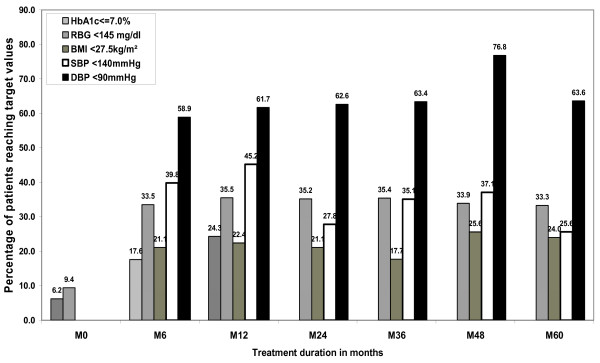
**Proportion of diabetic patients reaching the recommended optimal targets**. For body mass index (BMI), only patients with elevated baseline BMI (≥23.0) were included in the analysis. For systolic blood pressure (SBP), only patients with elevated baseline SBP (>130 mm Hg) were included in the analysis. For diastolic blood pressure (DBP), only patients with elevated baseline DBP (>80 mm Hg) were included in the analysis.

The proportions of patients (with baseline and follow-up values and not LTFU) reaching the recommended targets for glucose and BP are shown in Figure 2. In all, 24% (51/210) of patients had a HbA1c concentration below 7% and 35% (709/1,995) had an RBG less than 145 mg/dl within 1 year. In all, 45% (327/723) and 62% (373/605) of patients with systolic or diastolic hypertension at baseline, respectively, reached the treatment goals of ≥130/80 mm Hg within 1 year. As for weight control, only 22% (90/401) patients with obesity at baseline lowered their BMI below 27.5 kg/m^2 ^after 1 year.

**Figure 3:**
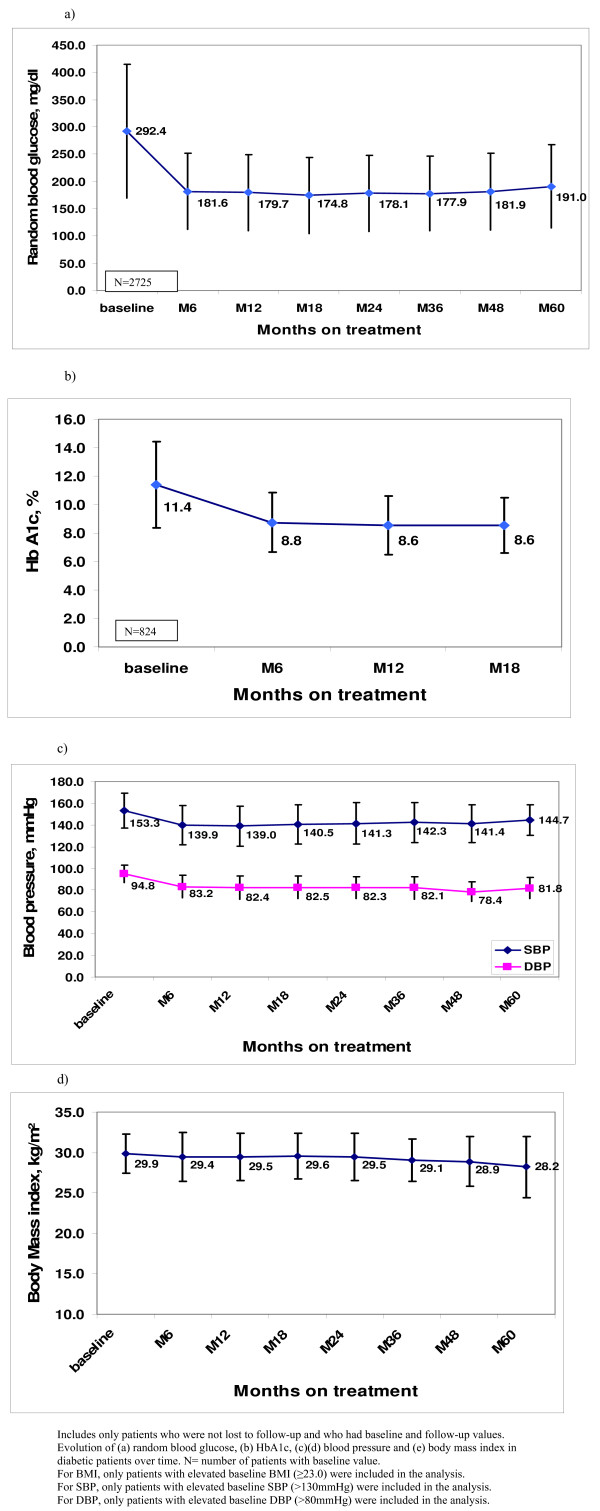
**Evolution of mean with standard deviation of biological markers in diabetic patients under treatment**. Evolution of **(a) **random blood glucose (RBG), **(b) **hemoglobin A1c (HbA1c), **(c, d) **blood pressure (BP) and **(e) **body mass index (BMI) in diabetic patients with baseline and follow-up values and not lost to follow-up. For BMI, only patients with elevated baseline BMI (≥23.0) were included in the analysis. For systolic BP (SBP), only patients with elevated baseline SBP (>130 mm Hg) were included in the analysis. For disastolic BP (DBP), only patients with elevated baseline DBP (>80 mm Hg) were included in the analysis.

Figure 3 illustrates the evolution of the mean values for RBG, HbA1c, BP and BMI over treatment time. Except for BMI, other parameters showed an initial drop during the first 6 months of treatment, which was maintained over the 5-year period. There was a significant drop of 109 mg/dl (95% confidence interval (CI) 103.1 to 114.3) in the mean RBG (*P *< 0.001) and a drop of 2.7% (95% CI 2.3 to 3.0) in the mean HbA1c (*P *< 0.001) between baseline and month 6 (Table 2). Among patients with hypertension at baseline, there was a drop of 13.5 mm Hg (95% CI 12.1 to 14.9) in the mean SBP (*P *< 0.001), and a drop of 11.7 mm Hg (95% CI 10.8 to 12.6) in the mean DBP (*P *< 0.001) between baseline and month 6.

**Table 2:** Mean differences between paired observations at baseline and month 6 in patients with baseline and follow-up values and not lost to follow-up

	Mean difference between baseline and month 6 (95% CI)*	*P *value for t test
RBG, mg/dl (n = 2,725)	108.7 (103.1 to 114.3)	< 0.001
HbA1c, % (n = 824)	2.7 (2.3 to 3.0)	< 0.001
SBP, mm Hg (n = 801)	13.5 (12.1 to 14.9)	< 0.001
DBP, mm Hg (n = 688)	11.7 (10.8 to 12.6)	< 0.001
BMI, kg/m^2 ^(n = 1,364)	-0.4 (-0.5 to -0.3)	< 0.001

For BMI, only patients with elevated baseline BMI (≥23.0) were included in the analysis. For SBP, only patients with elevated baseline SBP (>130 mm Hg) were included in the analysis. For DBP, only patients with elevated baseline DBP (>80 mm Hg) were included in the analysis.

*Mean difference between two observations taken on each individual.

BMI = body mass index; DBP = diastolic blood pressure; RBG = random blood glucose; SBP = systolic blood pressure.

### Risk factors for loss to follow-up

We included a total of 3,953 diabetic patients registered from 2002 until February 2008 who had attended at least 2 consultations in the analysis of risk factors for LTFU. We included all factors with a *P *value < 0.05 in the univariate analysis in the logistic regression model (Table 3). Factors significantly associated with LTFU were male gender, age older than 60 years, living outside the province, normal BMI (<23 kg/m^2^) on admission, high RBG (≥180 mg/dl) on last consultation, and coming late for the last consultation.

**Table 3:** Predictors of loss to follow-up (LTFU) among diabetic patients* (n = 3,953) by logistic regression analysis

Factors	LTFU cases/patients, (%)	OR	95% CI	*P *value	aOR†	95% CI	*P *value
Sex (n = 3,953):							
Male	430/1,332 (32.3)	1.0			1.0		
Female	731/2,621 (27.9)	0.81	0.70 to 0.94	0.004	0.79	0.67 to 0.93	0.005
Age, years (n = 3952):							
≥39	118/384 (30.7)	1.0			1.0		
40 to 49	242/1,056 (22.9)	0.67	0.52 to 0.87	0.003	0.72	0.54 to 0.98	0.034
50 to 59	389/1,395 (27.9)	0.87	0.68 to 1.11	0.27	1.02	0.77 to 1.36	0.878
≥60	412/1,117 (36.9)	1.32	1.02 to 1.69	0.03	1.54	1.15 to 2.05	0.004
Type of diabetes (n = 3953):							
Type 1	20/42 (47.6)	1.0			1.0		
Type 2	1,141/3,911 (29.2)	0.45	0.25 to 0.83	0.01	0.50	0.25 to 1.01	0.053
Origin (n = 3952):							
District of clinic	460/1,641 (28.0)	1.0			1.0		
Other districts	391/1,473 (26.5)	0.93	0.79 to 1.09	0.35	1.01	0.84 to 1.21	0.88
Outside province	310/838 (37.0)	1.51	1.26 to 1.80	<0.001	1.68	1.38 to 2.06	<0.001
Last BMI, kg/m^2^, (n = 3901):							
<23.0	581/1,708 (34.0)	1.0			1.0		
≥23.0	547/2,193 (24.9)	0.64	0.56 to 0.74	<0.001	0.70	0.60 to 0.82	<0.001
Last RBG, mg/dl (n = 3659):							
<180	330/1,688 (19.6)	1.0			1.0		
≥180	621/1,971 (31.5)	1.89	1.62 to 2.21	<0.001	1.81	1.54 to 2.13	<0.001
Last consultation (n = 3927):							
Not late	686/2,960 (23.2)	1.0			1.0		
Late	474/967 (49.0)	3.19	2.74 to 3.71	<0.001	2.72	2.29 to 3.24	<0.001

*Included are all patients who initiated treatment between March 2002 and February 2008 and who attended at least two consultations.

†All factors with *P *value < 0.05 in univariate analysis were included in the multivariable regression model. *P *value for the likelihood ratio test for the model was <0.001.

aOR = adjusted odds ratio; BMI = body mass index; CI = confidence interval; OR = odds ratio; RBG = random blood glucose.

## Discussion

The treatment outcomes of this large cohort of patients receiving standardized care in a resource-limited, chronic disease clinic setting with high prevalence of diabetes are encouraging. They support the feasibility of providing reasonably good care for large numbers of patients in developing countries. Overall, significant improvements in glycemia and BP were observed in patients within the first 6 months of treatment and were maintained throughout the study period. Nearly all patients had type 2 diabetes and were treated mainly with oral antidiabetic drugs. Unfortunately, although most patients improved, the majority did not reach the recommended targets for optimal diabetes control. Despite this, in light of the evidence that a decrease in glycosylated hemoglobin level is associated with a decreased risk for cardiovascular disease and mortality in persons with type 2 diabetes [5,6], patients with improved glycemia likely gained some clinical benefit even if they did not reach the target.

Other studies in both high and low resource countries have shown that the treatment targets for blood glucose in type 2 diabetic patients are difficult to reach in clinical practice. Large patient studies in industrialized countries have found relatively low proportions (24% to 36%) of diabetic patients with HbA1c below 7% [8,11,16]. Reliable data on glycemic control among diabetic patients treated in middle-income and low-income countries is more limited but no better. For instance, the goal for HbA1c below 7% was only reached by between 20% and 30% of patients treated in Thailand, in primary health care settings and at the tertiary hospital level, respectively [17,18], and by 46% of a cohort in Brazil [19]. In three Caribbean countries, 50% of patients had "poor" blood glucose control (≥10 mmol/l RBG) [20]. Results from small samples of patients in other low resource settings were similar: a median HbA1c of 8.5% was obtained in patients enrolled in a diabetic program in Eritrea [21], and 64% of patients showed poor control of HbA1c (>10%) over a 1-year period in Papua New Guinea [22]. In the Seychelles, less than a quarter of diabetic persons under treatment achieved recommended treatment target [23]. Although comparable to results from high-income countries, the quality of diabetes care in middle-income and low-income countries, from the limited findings published so far, is not optimal.

The reasons for disappointing optimal glucose control results in this setting are multifactorial and include poor adherence to taking medication, the use of RBG instead of HbA1c for patient monitoring, poor weight control, and lack of therapy intensification, such as insulin (<4% of patients on insulin). The very low number of patients on insulin in our study reflects the programmatic challenges of delivering insulin in a resource-constrained setting. In comparison, in the USA, around 30% of adults with diabetes are using insulin, either alone or combined with oral medication [24]. Barriers to the use of insulin in our setting include the financial cost, problems with cold chain storage, cost of and difficulties learning how to use glucose monitoring equipment, and less than ideal education and support for patient self-management. In a study in Brazil where 55% of type 2 diabetic patients were on insulin, problems in adjusting the insulin dose at home possibly led to poorer results among insulin-treated patients as compared to those using oral hypoglycemic agents [19]. Thus, although insulin is needed for therapy intensification, its proper use by patients remains challenging in resource-constrained settings.

With regard to hypertension, in industrialized countries it is estimated that only 30% of patients treated achieve their target BP goals, mainly due to poor adherence to treatment [25]. BP control was achieved in less than a quarter of diabetic persons under treatment in a poor African setting [23]. In light of these figures, the high proportion of patients with hypertension on admission who reached the BP targets was satisfactory in our program. However, we acknowledge that regression to the mean and habituation to repeated BP measurements could have had an effect on the decrease in BP observed during follow-up [26].

Weight control results in our diabetic cohort were poor and could be, in part, due to insufficient and inadequate patient education. Lifestyle changes imply both environmental and behavioral changes. In our context we cannot exclude a cultural reluctance to lose weight, since there is no dietary advice and no promotion of physical exercise at the population level in the country. This is compounded by the fact that in Cambodia, the rice-dominated diet is carbohydrate rich and most of the population living in urban and semi-urban areas tend to adopt a sedentary lifestyle. We suggest that the lack of adequate dietary advice and weight control could also have contributed to the persisting hyperglycemia observed.

The high LTFU observed among this cohort of diabetic patients contrasts with the low LTFU documented among HIV/AIDS patients treated in the same chronic disease clinic (3%) [12]. One contributing factor could be the inequality in care: HIV/AIDS patients received free health care, money for transportation, food, and social support, while diabetic patients did not get any of those benefits [27]. In our cohort, patients 60 years or older, patients late for their last appointment, and those living outside the province were more likely to be lost to follow-up. Older patients are unlikely to work and depend on family members for living and health expenditures, such as transport and drugs, over which they have no control. Another factor is that diabetic patients, contrary to HIV patients, have several alternatives for care such as traditional medicine and local pharmacies. Finally, the weakness of the counseling and patient education components of the diabetic care probably led to an insufficient understanding by patients of the disease and its consequences if untreated, leading to reduced motivation to continue treatment.

Although the integration of diabetic care within an HIV service allowed for a rationalization of resources [12], there were program costs related specifically to diabetic care that were not covered by the HIV program. For instance, in Cambodia HbA1c is only available in two locations and is at least eight times more expensive than CD4 count, used for monitoring HIV patients on antiretroviral therapy (ART). Additionally, other effective measures to reduce cardiovascular risks such as lipid-lowering drugs (statins) were beyond the program's financial capacity. However, the estimated cost for drug therapy based on the local drug prices was rather affordable at approximately US$48 per year for monotherapy (glibenclamide) and US$192 per year for bitherapy (glibenclamide and metformin). But overall, without free care offered by MSF, laboratory and drug costs, if assumed by patients, could act as barriers to meeting treatment goals.

This study has certain limitations. First, even though data were collected in standardized patient forms and entered prospectively into specifically designed software, errors or omissions could have occurred as data was collected from an operational program that was not designed specifically for research purposes. Second, we acknowledge that we only used biological measurements, such as glycemia and BP, as surrogate markers for diabetic outcomes. Additionally, the lack of standardized case definitions, access to specialized diagnosis and care for complications prevented us from collecting information about complications. Third, deaths were likely underestimated in our cohort and misclassified as LTFU, as we had no way of checking up on defaulters. The prognosis for diabetes in low-resource settings is poor for many patients as shown by the 5-year survival rates (60% to 84%) observed in diabetic patients in Africa [28]. Mortality surveillance and a defaulter tracing system were not in place in our program, as they required resources beyond our capacity. Fourth, our results only reflect treatment outcomes in diabetic patients attending care on a regular basis. By excluding patients LTFU in the analysis of treatment outcome, we introduced bias in the findings towards better results than there were in reality. Finally, until the last year of the program, we could only use RBG to estimate glycemic control instead of the gold standard, HbA1c, an expensive test and one not widely available test in our setting. Although a few studies have concluded that using RBG can be used to predict the quality of diabetic control in resource limited settings [13,29], we believe HbA1c provides a better measure and would encourage its use. Despite these limitations, our study's strengths were its large size, relatively lengthy follow-up, prospective data collection, and use of a specialized data software program.

## Conclusion

In conclusion, this study has shown that improvement in key biological markers can be obtained in large numbers of individuals with type 2 diabetes treated in a low-resource setting. Key features were a chronic disease structure to the program, standardized diagnosis and treatment protocols, multidisciplinary team, and heavily subsidized care. However, we learned that other interventions to further improve glycemic control should be pursued, including increasing the access to and use of insulin and HbA1c testing, and interventions to improve patient self-empowerment especially with regard to weight reduction. Complementary actions at the population level to promote lifestyle changes and achieve healthy body weight are also necessary components of good diabetic care and need to be established. These and other interventions in resource-poor settings require further operational research to improve care. However, our results do offer encouragement for the scaling up of care for chronic diseases such as diabetes, as they are a large and growing burden of illness in developing countries.

## Competing interests

The authors declare that they have no competing interests.

## Authors' contributions

MER participated in the design of the study, performed the statistical analysis, and wrote the draft manuscript. PI participated in the design of the study and in the program implementation, contributed to the interpretation of data, and was involved in the revision of the manuscript. TR and WVD participated in the design of the study, contributed to the interpretation of data, and were involved in the revision of the manuscript. CS participated in the diabetic care program implementation, contributed to the interpretation of data, and revised the manuscript. GA and LK contributed to the interpretation of data and revised the manuscript. All authors read and approved the final manuscript.

## Pre-publication history

The pre-publication history for this paper can be accessed here:


